# UK Dementia Trials Network: an expert‐informed framework for improving representation in dementia trials

**DOI:** 10.1002/alz.71928

**Published:** 2026-10-26

**Authors:** Igor C. Fontana, Tara Comber, Sheena Aurora, Daniel J Blackburn, Rachel F. Buckley, Christine Chow, Tiffany W. Chow, Ruth Croney, Sebastian J. Crutch, Karen Chilcott Holdridge, Lynne Hughes, Christopher Kipps, Susan Kohlhaas, Jorge Llibre‐Guerra, Paresh Malhotra, Charles R. Marshall, Stella McCaughey, Richard James Oakley, Rema Raman, Daniel Ridley, Shardae Showell, Richard Tsai, Catherine J. Mummery

**Affiliations:** ^1^ Department of Neurodegenerative Diseases Institute of Neurology University College London Queen Square, London UK; ^2^ Medical & Scientific Relations Alzheimer's Association Chicago Illinois USA; ^3^ Division of Neuroscience School of Medicine and Population Health Sheffield Institute for Translational Neuroscience (SITRaN), The University of Sheffield Sheffield UK; ^4^ Department of Neurology Massachusetts General Hospital, Harvard Medical School Boston Massachusetts USA; ^5^ Melbourne School of Psychological Sciences University of Melbourne Melbourne Victoria Australia; ^6^ Penn Neurodegeneration Genomics Center Department of Pathology and Laboratory Medicine University of Pennsylvania Perelman School of Medicine Philadelphia Pennsylvania USA; ^7^ Roche Products Ltd Welwyn Garden City UK; ^8^ Rare Dementia Support Centre, Institute of Neurology University College London, Queen Square London UK; ^9^ Eli Lilly and Company Indianapolis Indiana USA; ^10^ Global Alzheimer's Platform Foundation Washington District of Columbia USA; ^11^ Wessex Neurological Centre University Hospital Southampton NHS Foundation Trust Southampton UK; ^12^ Alzheimer's Research UK Cambridge UK; ^13^ Washington University in St. Louis St. Louis Missouri USA; ^14^ Department of Brain Sciences Imperial College London London UK; ^15^ Centre for Preventive Neurology Wolfson Institute of Population Health, Queen Mary University of London London UK; ^16^ Department of Neurology Barts Health NHS Trust London UK; ^17^ Atsena Therapeutics Durham North Carolina USA; ^18^ Alzheimer's Society London UK; ^19^ Alzheimer's Therapeutic Research Institute University of Southern California San Diego California USA; ^20^ Bristol Myers Squibb New York New York USA; ^21^ Biogen Inc Cambridge Massachusetts USA; ^22^ Denali Therapeutics South San Francisco California USA; ^23^ Dementia Research Centre, Institute of Neurology University College London Queen Square, London UK

**Keywords:** clinical trials, dementia, equity, representation

## Abstract

Alzheimer's disease (AD) is the leading cause of dementia worldwide, yet clinical trials remain unrepresentative of those most affected. Ethnic minoritized groups, socioeconomically deprived communities, adults over 75, and populations in low‐ and middle‐income countries are consistently underrepresented, limiting generalizability and reinforcing health inequities. This white paper draws on an international roundtable on Diversity and Inclusion in Dementia Trials, convened by the UK Dementia Trials Network at the Alzheimer's Association International Conference 2025. We determined that key barriers to research participation included geographic distance, financial burden, restrictive eligibility criteria, invasive procedures, and limited study partner support. We also highlight gaps in ethnic diversity and the inadequate integration of sex and gender considerations in trial design and reporting. Improving representation in AD trials is both a scientific and moral imperative. Achieving this requires coordinated action across community engagement and trust building, data transparency and reporting, global trial access and capacity building, eligibility criteria reform, and financial and logistical support for participants. We propose an expert‐informed implementation framework derived from the roundtable that identifies actionable strategies, responsible stakeholders, measurable indicators, and expected implementation outcomes to support translation of our recommendations into practice.

## Highlights

- Although progress towards more inclusive dementia trials has been made, achieving representative and equitable participation will require substantive systemic change. This depends on coordinated, sustained action across academia, industry, charities, and regulators, who must be jointly accountable for delivering improvements.
- Sustained, community‐centered engagement is essential to build trust and support participation among underrepresented groups. This can be achieved through long‐term partnerships with trusted local and faith‐based organizations, diverse research teams, and co‐developed culturally relevant materials.
- Representation should be recognized as a core indicator of research quality and embedded across trial design, analysis, and reporting. Transparent, consistent reporting of disaggregated demographic data is essential to ensure accountability and improve the generalizability of trial findings.
- Reducing the practical burdens of participation is critical to improving equitable access. Research sites and sponsors should provide fair reimbursement for travel and caregiving costs, offer flexible scheduling, and incorporate decentralized or hybrid models to reduce participant burden.
- Trial design and eligibility criteria must be re‐evaluated to avoid systematically excluding diverse populations. Adopting culturally neutral assessment tools and reviewing non‐essential exclusions would improve representation without compromising safety.
- The existence of long‐term partnerships between HICs, LMICs, and industry can stimulate workforce development and provide support/mentorship to empower the development of trial networks in LMICs.
- Work is needed to ensure equitable and scientifically robust trial design by fully integrating biological variables such as sex across study design, statistical powering, outcome reporting, and data transparency. Key recommendations include routinely reporting sex‐stratified outcomes and releasing disaggregated data to enable secondary analyses of sex differences.

## 1 INTRODUCTION

Alzheimer's disease (AD) is the most common cause of dementia and has a major global impact, estimated to affect 55 million people around the world in 2024.^1^ Progress toward an effective treatment or cure for AD is dependent on the rigor of clinical trials and generalizability of results by including a representative global population.^2^ Despite this, rural, socioeconomically deprived, and racial and ethnic minoritized populations, including Black, Hispanic, indigenous populations, and Asian groups, remain markedly underrepresented in AD trials.^2^, ^3^ Participation rates among these populations are often substantially lower than their regional disease prevalence and, even when included, too small to permit statistically powered subgroup analyses.^4^ Consequently, the role of race and ethnicity in treatment efficacy and safety cannot be adequately assessed, including distinct biomarker signatures, drug metabolism, and treatment response, limiting the generalizability of trial outcomes and perpetuating existing health disparities.^3^, ^5^, ^6^ Such gaps in representation reflect broader structural inequities in global research, extending beyond racial and ethnic dimensions to geographic and socioeconomic disparities.^2^, ^7^ Clinical trial sites are fielded more commonly in high‐income countries (HICs), with less than 12% conducted in low‐ and middle‐income countries (LMICs), despite LMICs’ growing dementia burden.^2^ Furthermore, within HICs, clinical trials are concentrated in economically privileged areas with established research infrastructures, leading to consistent underrepresentation across these key groups and inequality of research outcomes.^8^

To begin to address inequity in clinical trial availability or eligibility, the National Institute for Health and Care Research (NIHR)‐funded United Kingdom Dementia Trials Network (UKDTN, www.ukdtn.org) hosted a roundtable on diversity in dementia trials at the July 2025 Alzheimer's Association International Conference (AAIC) in Toronto, bringing together leadership from the industry sector, international academic institutions, and charities. The discussion led to suggested priorities for making trial designs more inclusive, best practices for community engagement, and structural changes to promote equitable access to dementia research worldwide with focus on those with lived experiences.

## 2 UKDTN ROUNDTABLE

This white paper was developed following a 120‐min roundtable convened by the UKDTN during AAIC 2025. The roundtable included 23 participants (Supplemental Table 1) invited by the UKDTN team based on their expertise or role in dementia clinical research, patient and public involvement, industry, or research infrastructure to ensure representation across stakeholder groups. The perspectives of people living with dementia (PLWD) and care partners were incorporated through a UK‐wide survey conducted by Alzheimer's Society and the UK Dementia Trials Network alongside contributions from dementia charities and organizations (Alzheimer's Association, Alzheimer's Society, Alzheimer's Research UK), six pharmaceutical and biotechnology companies, and 10 academic institutions. Several participants had experience collaborating with or leading research in LMIC settings, providing perspectives relevant to LMIC research contexts in addition to the HIC context. This composition was intended to bring together complementary perspectives relevant to the design, implementation, representation, and delivery of dementia clinical trials.

Invited panelists delivered 10‐min presentations on their areas of expertise (Table 1), followed by a discussion that included all participants, facilitated by Professor Tiffany Chow. Recommendations were identified and extracted from that discussion in agreement, then refined by the authors using audio transcripts generated during the session. Although recommendations were not formally ranked or scored, they reflected the key issues and priorities emerging across the presentations and moderated discussion. These recommendations were honed through iterative review by the authors, comprising panelists and other participants in the roundtable. Where differences in interpretation or emphasis arose during the iterative review process, these were resolved through discussion until agreement was reached. All co‐authors reviewed and endorsed the final recommendations, which are summarized in Table 2 and discussed in the subsequent sections.

**TABLE 1:** Roundtable panelists and areas of expertise in clinical trials.

Panelist	Affiliation	Area of expertise / focus
Sebastian Crutch	University College London, UK	Patient experience and perceptions of clinical research and trials
Jorge Llibre‐Guerra	Washington University School of Medicine, USA	Geographical inequities in clinical trials
Rachel Buckley	Harvard Medical School, USA and University of Melbourne, Australia	Sex differences in clinical trials
Charles Marshall	Queen Mary University of London, UK	Structural issues in clinical trials
Shardae Showell	Biogen Inc.	Industry diversity and inclusion efforts in clinical trials
Kathryn Stacey	Roche Products Ltd.	Inclusion in clinical trials

**TABLE 2:** Implementation framework to improve diversity in dementia trials derived from the UKDTN International roundtable.

Domain	Challenges identified	Level of implementation	Actionable strategies	Responsible stakeholders	Implementation indicators and expected outcomes
**1. Community engagement and trust building**	Study‐specific outreach fails to build trust and retention in underrepresented communities	Study/site, institutional, community	Partner with local community and faith‐based organizations Employ culturally and linguistically diverse recruitment staff and cultural liaison officers at research sites Use mobile research units to reach remote/rural areas (only if combined with other elements) Co‐develop culturally tailored outreach and consent materials with people with lived experience and community partners Embed engagement metrics in institutional and trial performance reviews Require evidence of engagement in study approval processes	Industry, academia, Charities, regulators	**Indicators**: Number/proportion of studies with: Documented partnerships with local communities Culturally and linguistically diverse recruitment staff or cultural liaison support, where appropriate Community co‐developed outreach and consent materials Documented engagement metrics incorporated into trial/institutional performance reviews or study approval processes Use of mobile/decentralized strategies to reach remote or rural populations Screening/enrollment ratio Recruitment/retention rates across demographic groups Reasons for screen failure and study discontinuation, including demographic or socioeconomic differences where available Participant‐reported experience, trust, and study burden Representation of enrolled participants relative to population the trial is intended to serve **Expected outcomes**: Improved recruitment and retention Improved participant experience, trust, and engagement Greater representation of populations relevant to trial More consistent and accountable implementation of community‐engagement strategies
**2. Data transparency and reporting**	Lack of disaggregated demographic data limits secondary analyses and perpetuates bias	Study, registry, publication, institutional	Standardize collection and reporting of race, ethnicity, geography, sex, and socioeconomic variables Publicly release anonymized, disaggregated datasets Develop open‐science mandates through funders and charities Require subgroup reporting as a condition for trial registration/publication	Industry, academia, charities, regulators	**Indicators**: Proportion of participants with complete demographic data Proportion of trials reporting prespecified demographic variables and subgroup outcomes Proportion of eligible datasets made publicly accessible in anonymized, disaggregated form Number/proportion of funders and charities adopting open‐science or data‐sharing requirements Proportion of trials meeting applicable demographic reporting and data‐sharing requirements for registration/publication Frequency of secondary use of publicly accessible trial data **Expected outcomes**: Improved completeness and transparency of demographic data Increased availability and reuse of disaggregated trial data Improved reproducibility and accountability Greater ability to assess equity and heterogeneity of trial outcomes
**3. Global trial access and capacity building**	Concentration of dementia trials in HICs limits representation of populations in LMICs and other underserved regions; inadequate infrastructure, workforce capacity and regulatory pathways constrain sustainable trial delivery	Regional/national, institutional/site, health‐system, regulatory	Expand clinical trial networks into LMICs and historically underserved regions Invest in local research infrastructure, workforce development, and investigator training Establish long‐term, equitable HIC‐LMIC‐industry partnerships Strengthen local regulatory and ethics‐review capacity and streamline trial approval pathways Align trial activity with local healthcare and public health priorities Develop sustainable regional trial networks and shared clinical/biological data resources Consider post‐trial access to successful interventions when designing international trials	Industry, academia, governments, regulators, charities, funders	**Indicators**: Number/proportion of trials and sites in LMICs Local investigator leadership and workforce trained Number/proportion of studies investing in local research infrastructure and sustainable regional trial networks Regulatory/ethics approval timelines, participant representation relative to local populations Proportion of studies with equitable local partnerships and post‐trial access plans **Expected outcomes**: Increased access to dementia clinical trials in LMICs Strengthened and sustainable local research capacity and infrastructure More equitable and durable international research partnerships Improved representation and generalizability of trial findings across global populations Improved alignment of research with local healthcare and public health priorities
**4. Eligibility criteria reform**	Overly restrictive criteria exclude individuals with comorbidities most prevalent in underrepresented population(s), limiting generalizability	Protocol/study, regulatory, institutional	Systematically review and justify each exclusion criterion Eliminate exclusions not essential to scientific validity or safety Include participants with common comorbidities (e.g., diabetes, cardiovascular disease) after providing means to better control the conditions Encourage adaptive trial designs allowing stratification by comorbidity Review biomarker‐based eligibility criteria Address culturally sensitive cognitive assessments	Industry, academia, regulators	**Indicators**: Proportion of exclusion criteria with documented scientific/safety justification Proportion of screened participants excluded due to comorbidity or biomarker thresholds Demographic/comorbidity profile of enrolled participants Proportion of protocols incorporating stratification and other inclusive design approaches to accommodate relevant comorbidities, adapting biomarker‐based eligibility, using culturally sensitive cognitive assessments **Expected outcomes**: Broader and more inclusive eligibility criteria Increased participation of people with common comorbidities and diverse backgrounds Reduced exclusion attributable to non‐essential eligibility criteria Improved representativeness and external validity of trial findings
**5. Participant support: financial and logistical**	Travel, caregiving, and opportunity costs deter participation; reimbursement inconsistently applied	Study/site, institutional, regulatory	Implement standardized, timely reimbursement procedures for travel and caregiving costs Define reimbursement as an equity measure, not undue inducement Establish regulatory guidance normalizing fair compensation Adopt decentralized or hybrid trial models to reduce participant burden Address study‐partner requirements	Industry, academia, regulators	**Indicators**: Proportion of participants receiving reimbursement and time from expense to reimbursement Participant‐reported financial/logistical burden Recruitment/retention by socioeconomic/geographic characteristics Use of decentralized/hybrid approaches Proportion of trials receiving required study‐partner support Adoption of regulatory or institutional guidance supporting fair participant compensation and reimbursement Participant‐reported satisfaction with reimbursement and support arrangements **Expected outcomes**: Reduced participation burden Timely/consistent reimbursement processes Improved equitable access and retention of trial participants
**Cross‐cutting principle**	Diversity often treated as ancillary goal	Program, institutional, study, policy	Elevate inclusion metrics as measures of research quality Incorporate equity outcomes into trial success criteria	All stakeholders	**Indicators**: Proportion of trials with: Prespecified diversity/equity targets and monitoring plans Reporting of recruitment, retention, and outcomes by relevant demographic groups Incorporation of equity metrics into governance and trial evaluation **Expected outcomes**: Diversity and equity become integral to research quality Equity incorporated into trial design and evaluation Improved scientific validity, representation, and ethical conduct of clinical trials

*Note*: Recommendations presented in Table 2 were derived from themes identified during roundtable discussions and subsequently refined through iterative review by all co‐authors.

The UKDTN international roundtable included a broad range of stakeholders; however, it was not intended to provide exhaustive representation of all geographic regions, healthcare systems, or groups within the dementia research ecosystem. Therefore, the recommendations presented in this manuscript should be interpreted as expert‐informed priorities arising from the perspectives represented at the meeting rather than as a comprehensive global consensus.

## 3 PARTICIPANTS’ EXPERIENCES IN DEMENTIA RESEARCH

Tackling dementia has become a matter of the highest international priority and requires urgent action. In the UK alone, over 70,000 people died due to dementia in 2025, making it the leading cause of death. To inform discussions around participation and representation in clinical trials, the Alzheimer's Society and UKDTN conducted a survey of PLWD and carers regarding their experiences, awareness, and perceptions of dementia research. The survey was designed to provide insights into participant experiences rather than nationally representative estimates.

The anonymous online survey, co‐created with healthcare professionals and people with lived experience, was disseminated through clinical networks, National Health Service (NHS) sites, the Alzheimer's Society website and social media, patient support groups, and services between February and April 2025. As the survey was disseminated through open networks and platforms, a denominator was unavailable and a conventional response rate could not be calculated. Detailed methodology and analyses will be reported separately. As individuals chose to self‐report, there are clear potential biases; however, this was meant to provide a baseline indicator rather than a strictly representative sample. Preliminary analysis from 1202 participants (24% PLWD and 76% carers responding on behalf of those they care for) showed that only 18% (*n =* 219) of respondents had previously participated in clinical trials or other type of dementia research. In addition, 93% identified as White, highlighting a substantial lack of ethnic diversity in dementia research participation. Similar underrepresentation of ethnic minoritized groups has been documented across other areas of clinical research, including cancer.^9^, ^10^ Limited representation may also restrict the generalizability of trial findings to the broader populations who may ultimately receive an intervention.^11^ Therefore, we acknowledge that this group may not be representative, given the self‐selected nature of the survey; nonetheless, these findings provided a baseline indicator of experiences and perceptions of dementia research rather than nationally representative estimates.

Among those who had participated in research, the most common motivations for taking part, as described by survey respondents, were wanting to make a difference to others, wanting access to new treatments or devices, seeking a sense of purpose and control, and access to healthcare professionals. Conversely, among those who had been approached about research but could not take part, the most commonly reported barriers were distance to the research center, travel costs associated with participation, not wishing to have additional lumbar punctures, not being able to commit to the study schedule, feeling nervous about taking part in a study with an experimental drug and its possible side effects, and lack of an appropriate study partner. These findings highlight the fact that equitable participation requires attention not only to willingness to participate but also to the practical, financial, and logistical conditions that enable people to do so.

In terms of general opinions about dementia research, 96% felt that clinical trials and other forms of dementia research were an important part of improving the lives of PLWD, 94% felt more was needed to raise awareness of dementia clinical trials and other forms of dementia research, and 91% felt that clinical trials and research should be discussed as a core part of dementia treatment and care. Together, these findings highlight strong public recognition of the importance of dementia research and a clear expectation that clinical trials should be embedded within routine dementia care. However, this support and perceived importance are not yet matched by equitable access to, or representation within, dementia clinical trials worldwide, highlighting the need for sustained community engagement and accessible routes into research participation (Item 1, Table 2).

## 4 GLOBAL DISPARITIES IN CLINICAL TRIALS

For most causes of dementia, including AD, aging is the greatest risk factor posing significant challenges for public health systems, economies, and social support structures. Accounting for over 80% of the global population, LMICs are projected to become aging societies by 2053, when the population ≥60 years will surpass all other age groups in size. Despite this, LMICs remain substantially underrepresented in the development and evaluation of dementia trials. Approximately 1237 AD and related dementias (ADRD)–modifying therapies have been tested across 74 countries, resulting in 3467 clinical trials. Of these, 3065 trials (88.4%) were conducted in HICs, while 405 trials (11.6%) took place in emerging economies and LMICs.^2^ This stark disparity highlights a significant gap in research investment and access to innovative therapies for populations in LMICs, where the burden of aging is rapidly increasing.^12^

Multiple barriers continue to limit the expansion of clinical trials in LMICs. These include underfunded research infrastructure, shortages of trained clinical trial personnel,^13^ and fragmented regulatory pathways that can delay study initiation.^14^ Ethics review processes are often resource‐constrained, resulting in slower approval timelines, while the absence of established reimbursement mechanisms for diagnostics, trial‐related procedures, and eventual therapies may reduce incentives for healthcare systems to support research participation.^15^, ^16^ In some settings, political instability, economic uncertainty, and competing public health priorities further restrict investment in research,^17^ halting the development of sustainable dementia clinical trial networks. Additional concerns include uncertainty regarding post‐trial access to successful therapies^18^ and pharmaceutical industry perceptions of limited commercial viability,^19^ which may discourage investment despite the substantial and growing disease burden in these regions. Addressing these structural disparities therefore requires streamlining regulatory processes, developing sustainable funding mechanisms, and promoting transparent data generation and sharing (Item 2, Table 2).

Global collaboration between HICs and LMICs is essential to advance ADRD clinical trial research. To achieve this, substantial investment in research infrastructure and research‐oriented higher education institutions is required to build sustainable local research capacity (Item 3, Table 2). Additionally, governments should consider implementing regulatory reforms aimed at reducing approval timelines and expediting clinical trial processes to attract greater funding and participation. Regional trial platforms currently operating in LMICs, such as DIAN‐TU_LatAm,^20^ API_Colombia,^21^ and LatAm‐FINGERS,^22^ demonstrate alternative strategies for overcoming structural and logistical barriers. These initiatives highlight potential solutions to increase representation from diverse populations in ADRD clinical trials and to expand equitable access to therapeutic research across LMIC settings and act as pioneers in the development of more equitable trials. The existence of long‐term equitable partnerships between HICs, LMICs, and industry can stimulate local workforce development, train local investigators, align with local public health priorities, drive consideration of post‐trial access, and establish sharable clinical and biological data resources (Item 3, Table 2). These partnerships can provide support and mentorship to empower the development of trial networks in LMICs. This fosters non‐extractive global research in a sustainable manner building up infrastructure for future ADRD trials.

## 5 EQUITABLE AND REPRESENTATIVE TRIAL DELIVERY

The concentration of dementia clinical trials in HICs results in unequal access to research and limits the generalizability and applicability of trial outcomes. It is important to note that representation and equity should not be used interchangeably. Representation refers to the demographic characteristics of enrolled trial populations, whereas equity concerns whether underrepresented groups have comparable opportunities to participate, meet eligibility criteria, remain enrolled, and finally benefit from clinical trial outcomes.

In the context of an uneven global distribution of risk factors, as well as genetic, racial, and socioeconomic diversity across regions, unequal access and poor representation of diverse populations hinder the relevance of findings for LMICs and HICs. A 2022 review by Franzen et al. found that 95% of participants in 101 Alzheimer's trials (2001 to 2019) were White, with no improvement over the 18‐year period studied.^4^ More recent phase 3 lecanemab (CLARITY‐AD) and donanemab (TRAILBLAZER‐ALZ 2) trials showed similar disparities, with 80% to 90% of participants White and only around 2% Black participants receiving active treatment.^23^, ^24^ In CLARITY‐AD and A4, a sharp drop in Black participants between screening and randomization suggests that eligibility criteria may disproportionately exclude these populations.^3^, ^23^ Overcoming these disparities also requires consistent collection, disaggregated reporting and transparent sharing of demographic and trial outcome data, allowing differences in recruitment, retention, eligibility, and treatment response to be identified and addressed (Item 2, Table 2).

Inclusive trial design is essential to ensure that eligibility criteria and assessment requirements do not unnecessarily exclude populations who are likely to receive the intervention in routine practice. Trial designs often apply rigorous eligibility criteria that exclude individuals with common comorbidities such as diabetes, cardiovascular disease, and mood disorders, which may be more prevalent in populations already underrepresented in trials.^25^ This limits the generalizability of findings across populations and hinders clinicians’ ability to make informed decisions about how best to treat patients in real‐world settings. In addition, comorbidity prevalence varies with population, leading to unequal exclusion from trials.^26^ Beyond comorbidities, age is also a limiting barrier in dementia clinical trials with those above the age of 75 often underrepresented, despite being the most affected by the condition.^27^ Inclusion/exclusion criteria should be carefully reviewed to ensure each item is scientifically justified, removing barriers for underrepresented populations (Item 4, Table 2).

Apart from biological factors, structural and social determinants of health drastically impact clinical trial design and outcomes. Screening tools (e.g., Mini‐Mental State Examination), which are culturally and educationally biased,^28^, ^29^, ^30^ further exacerbate the exclusion of underrepresented groups. More ecologically valid measures that focus on everyday function, rather than abstract cognitive tasks influenced by education, language, or literacy, may offer a fairer and more representative assessment of disease severity (Item 4, Table 2). However, while culturally adapted or ecologically valid assessments may improve inclusivity, such instruments must demonstrate measurement equivalence, validity, reliability, and responsiveness across populations before implementation in registrational trials. Ensuring psychometric robustness is essential to avoid introducing new sources of bias while addressing existing limitations of traditional cognitive assessments.

Trial site selection can also act as another structural barrier to equitable and representative participation. Clinical trials are typically run in large, highly resourced university hospitals or private centers, which may be less accessible to underserved communities facing geographic, socioeconomic, and logistical barriers.^31^, ^32^ The embedding of research in clinical centers improves the quality of clinical care,^33^ so limited access of underrepresented groups not only hinders trial participation but also deprives them of access to higher standards of care.^34^ One example of the importance of leveraging community relationships is provided by the Alzheimer's Association, which successfully recruited a diverse cohort in the U.S. POINTER study, with over 30% of the 2100 older adult participants drawn from ethnoracially underrepresented groups.^35^ Community‐based sites can reduce travel burdens and improve access by fostering more welcoming environments. Nevertheless, it is fundamental that resources are allocated to training healthcare professionals to staff these sites, ensuring that the highest level of care is delivered within the community (Item 1, Table 2).

Participant support is another essential component of equitable trial delivery. Trial participation can create economic burdens, particularly for low‐income populations with limited flexibility to take time off work, especially in studies enrolling younger adults.^36^ This constraint is further compounded by the requirement in many trials that participants attend visits with an informant or study partner (Item 5, Table 2), who must also adjust their work schedules. While this is the case to some extent globally, it is particularly problematic in LMIC populations. Fair and prompt remuneration and support with travel costs are equally important to remove financial and logistical barriers to participation.^37^, ^38^ Working together, industry and academia can introduce standardized, timely reimbursement systems covering travel and caregiving costs, building decentralized trial models to reduce participant burden.^39^ To enable this, reimbursement should be structured in accordance with applicable ethical and regulatory requirements to avoid undue influence or coercion, thereby normalizing compensation as part of good trial practice (Item 5, Table 2).

Legal protection against workplace repercussions for time spent in clinical trials may also be necessary. Trust is also central to participant engagement: Building on long‐standing community relationships is far more effective than ad hoc outreach for a specific study, which can feel exploitative or intrusive. Greater representation of diverse groups among trial staff may also help build trust and facilitate participation, as supported by previous evidence showing that individuals are more likely to participate in research when approached by someone of the same race or ethnic background.^40^ These approaches highlight the importance of sustained community engagement and trust building as core components of equitable trial delivery (Item 1, Table 2).

## 6 SEX DIFFERENCES IN CLINICAL TRIALS

Sex and gender are critical determinants of disease incidence, progression, and outcomes. Numerous conditions such as AD, cardiovascular disease, cancer, and COVID‐19 exhibit differences in prevalence rates and biological mechanisms between sexes that could impact the generalizability of clinical trial safety and efficacy findings. These disparities highlight the necessity of integrating sex‐based analyses. Historically, clinical trials have disproportionately enrolled male participants, with a direct impact on understanding sex‐specific drug effects. Between 1997 and 2000, 10 drugs were withdrawn from the US market,^41^ and eight of these withdrawals were attributed to higher risks and adverse effects in women. Notably, approximately 80% of these drugs had been tested predominantly in male cohorts. The consequences of excluding sex‐specific considerations in drug development and safety evaluation are significant.

While progress has been made, further efforts are required to ensure equitable and scientifically rigorous trial design. Building on established evidence and consensus recommendations, including those outlined by the National Academies of Sciences, Engineering, and Medicine (NASEM) on improving representation in clinical trials and research,^42^ incorporating sex as a biological variable should extend beyond recruitment strategies to encompass study design, statistical powering, outcome reporting, and data transparency (Item 2, Table 2). In fact, key recommendations include the routine reporting of sex‐stratified outcomes and the release of disaggregated data to facilitate secondary analyses focused on sex differences.^43^ Moreover, clinical trials should be adequately powered to evaluate sex as a crucial dependent factor of the primary endpoint. Although women now often represent most AD trial participants, this bias must be interpreted with caution and contextualized within broader demographic, higher risk, and disease‐specific frameworks. Enhancing diversity within research teams, including representation across sex, age, and other sociodemographic factors, is also essential to ensure inclusive and generalizable findings.

Sex differences in AD are well documented and may impact treatment efficacy and trial outcomes. Females have a higher lifetime risk of AD compared to males and typically exhibit greater amyloid and tau burden as well as faster rates of brain atrophy.^1^, ^44^, ^45^, ^46^ These biological variations may influence pharmacological response, with subgroup analyses of lecanemab in the pivotal CLARITY‐AD trial indicating potential differences in clinical benefit between men and women^47^; however, donanemab did not show the same effect – highlighting the need for examining sex differences as a priority. It is also essential to consider how gender differences influence disease and trial outcomes. Factors closely linked to gender such as education, occupation, and lifestyle are established determinants of cognitive resilience and AD presentation.^48^, ^49^ These social and behavioral factors interact with biological sex, which further highlights the need for adequately powered sex‐ and gender‐stratified analyses and transparent reporting of disaggregated data in dementia trials. In conclusion, integrating sex and gender considerations into clinical trial methodology is not merely a matter of equity; it is a scientific imperative that enhances the validity, safety, and applicability of biomedical research (Item 2, Table 2).

## 7 INDUSTRY PERSPECTIVES ON INCLUSION

Industry sponsors are committed to improving strategies for recruitment, screening, and retention of underrepresented populations in dementia trials. At the UKDTN international roundtable, representatives from Biogen and Roche shared insights into how industry is addressing underrepresentation in dementia clinical trials with a particular emphasis on inclusive trial design. All participant companies at the roundtable acknowledged the critical importance of ensuring that the demographics of trial participants reflect the populations who will ultimately receive the treatments.

To strengthen community engagement, enhance transparency, and reduce participation barriers, Biogen has launched a range of initiatives. These include clinical development‐led webinars, site visits, mobile research units (MRUs), and strategic partnerships with trial sites that have the staff and infrastructure to support diverse populations. At AAIC 2024, Biogen presented data showing that these coordinated efforts successfully increased screening rates and expanded outreach within diverse communities; however, enrollment outcomes continued to be challenged by disproportionately higher screen failure rates among African American and Hispanic populations in the United States.^50^

Given the higher prevalence of AD in underrepresented populations, Biogen analyzed screen failure data to better understand underlying drivers. Their findings showed that this higher prevalence reinforced cycles of socioeconomic inequality, limiting access to clinical trials and, prospectively, to emerging therapies. Biogen recently presented new findings on “infusion deserts”^51^ – defined by Biogen as locations where people lack reasonable access to medical facilities providing essential IV infusion treatments – highlighting how geographic and structural barriers further disadvantage vulnerable groups and pointing to the need for sustained efforts to close research and access gaps, including novel delivery methods (e.g., home infusions) and exploration of less invasive ways of administering therapies.

An expanding area of dementia research is in gene silencing; the first‐generation therapies require intrathecal delivery. This method delivers treatment directly to the central nervous system, potentially bypassing systemic effects associated with comorbidities that disproportionately affect certain populations. However, intrathecal administration may present challenges related to post‐approval scalability and access. Different companies are investigating alternative delivery methods for large‐molecule therapies such as oligonucleotides (e.g., BIIB080), for example, implanted devices and IV, and subcutaneous formulations using active transport mechanisms to cross the blood‐brain barrier. This next generation of delivery methods may offer more practical and inclusive solutions for diverse populations, with potential to provide easier delivery, but also reduced dosage, and hence reduced side‐effect potential, and more rapid effect, hence shorter treatment periods.

Roche has similarly prioritized inclusive trial design and works hand in hand with patient organizations and people with lived experiences. Patient and public involvement (PPI) is sought to ensure robust, transparent processes, and meaningful communication can be provided to study participants. This has led to multiple initiatives being implemented in recent trials. Inclusion and exclusion criteria are reviewed to ensure that each item is scientifically justified, and when designing the schedule of assessments, more consideration has been given to optimizing frequency visit length and reducing invasive and burdensome procedures on participants, as well as increasing flexibility by allowing remote assessments for carers, in support of making trial participation more accessible.

​​ Using data‐driven analytics, Roche actively seeks trial sites in regions that are more likely to include diverse and underserved populations​.​​ Additional strategies ​​include​​ leveraging Roche's Advancing Inclusive Research (AIR) site alliance, incorporating sites already engaged in AIR partnerships with Roche across other therapeutic areas. In the UK specifically, ​50% of the sites taking part in TRONTIER 1 and 2 (NCT07169578 and NCT07170150) were intentionally selected to support inclusive research goals through existing inclusive research initiatives and catchment areas with greater than the national average of ethnic minority residents and deprived households.​​​

In the United States, this strategic site selection is complemented by the deployment of MRUs, which enter rural and underserved communities directly. These units reduce the need for participants to travel to clinical sites and help raise awareness of clinical trials among populations with limited prior exposure to research participation. Importantly, the use of MRUs is most effective when embedded within broader, long‐term community relationship‐building efforts – supporting not just trial‐specific visits but ongoing engagement, trust, and visibility. This successful example could be replicated in other countries. For instance, the UK has increasing access to MRUs; therefore, this model can similarly be leveraged across the country to strengthen sustained brain‐health community initiatives and facilitate trial pre‐screening within the context of deeper, continuous partnerships.

​​ A key initiative is the TRAVELLER pre‐screening study (NCT07177352), which uses a brief clinical assessment and a plasma biomarker (Elecsys pTau217) to identify​ ​potentially eligible participants​ with low burden and can offer ​an introduction​ to clinical trials​. This approach enables broader community outreach and extends trial access to populations that more accurately reflect the epidemiology of AD. Similar initiatives, such as the Alzheimer's Prevention Trials Webstudy and AlzMatch,^52^​​ also aim to facilitate the pre‐screening and identification of potential participants to access AD clinical trials.

​​​ Site‐enabling activities are also a key focus to ensure the efficient use of site resourcing and supporting infrastructure, for example, implementing trial‐agnostic rater training and upskilling and imaging qualification.​​ In addition, developed in collaboration with the Alzheimer's Society and the UKDTN, patient entry and exit questionnaires have been incorporated into TRONTIER 1 and 2 trials in order to gain a deeper understanding of participatory experiences and to further understand expectations for both trial participants and their care partners.

Both Biogen and Roche acknowledged the importance of partnering with trial sites and supporting their efforts to improve representation. They also highlighted the value of collaborations with community groups, including faith‐based groups and non‐profits, which play a critical role in building trust and fostering sustained engagement with underrepresented communities (Item 1, Table 2).

## 8 DISCUSSION

Persistent underrepresentation of racially, ethnically, socioeconomically, and geographically diverse populations in AD clinical trials challenges both generalizability and equity in access to emerging therapies. Addressing this imbalance requires coordinated action across industry, academia, charities, and regulators to improve community engagement, data transparency, participant support, and eligibility practices. The recommendations from the UKDTN international roundtable at AAIC 2025 build on previous efforts to improve equity and representation in clinical research, including the 2022 NASEM consensus report on improving representation in clinical trials and research^42^ and, more recently, the World Health Organization guidance on equitable clinical trials.^53^ Building on these principles, the UKDTN implementation framework (Figure 1) focuses on barriers that are particularly relevant to dementia clinical trials, including study partner requirements, biomarker‐based eligibility criteria, culturally sensitive cognitive assessments, and the substantial burden placed on older participants and their caregivers. It distinguishes between system‐level barriers and the actions required to address them across stakeholders (Table 2).

**FIGURE 1:**
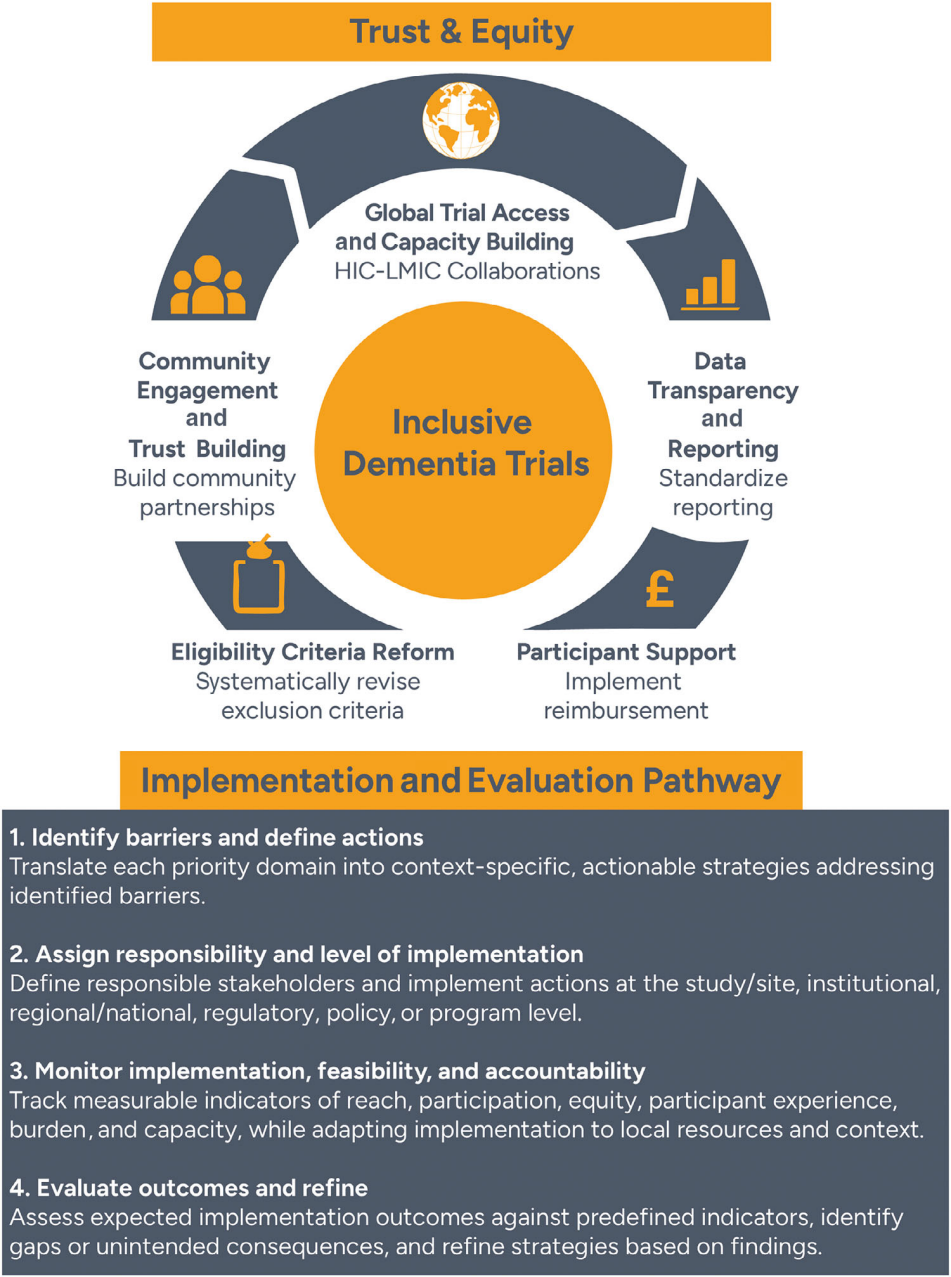
Implementation and evaluation framework for inclusive dementia trials. The framework comprises five priority domains: community engagement and trust building, global trial access and capacity building, data transparency and reporting, eligibility criteria reform, and participant support. The implementation and evaluation pathway outlines four sequential steps: identifying barriers and defining actions; assigning responsibility and implementation level; monitoring implementation, feasibility, and accountability; and evaluating outcomes and refining strategies. Indicators may assess reach, participation, equity, participant experience, burden, and capacity across relevant implementation levels.

Implementation strategies include strengthening community‐based recruitment through partnerships with community organizations, including faith‐based groups; employing diverse recruitment staff; embedding recruitment teams within community infrastructure; using cultural and bilingual liaison officers; and deploying mobile units to reach rural populations. Participant engagement should also be incorporated into site performance assessment using relevant engagement metrics. Finally, co‐development of culturally relevant materials and outreach approaches with people with lived experience, trial sites, and industry partners can ensure that recruitment information will be accessible and responsive to community needs (Item 1, Table 2).

Expanding trials beyond the Global North will enable the study of AD phenotypes across diverse genetic, environmental, lifestyle, and socioeconomic contexts, improving the applicability of findings across populations. This increased heterogeneity will strengthen the generalizability of treatment effects, improve external validity, and address long‐standing gaps in the representation of people from ethnic minoritized backgrounds and LMICs (Item 3, Table 2).

Data transparency is another cornerstone of equitable research. The lack of disaggregated data on race, sex, geography, and socioeconomic status limits secondary analyses and perpetuates bias. Industry and academia should commit to standardized reporting and public release of anonymized, disaggregated datasets. Just as charities advocate for open‐science mandates, regulators can advance transparency by requiring subgroup reporting as a condition of trial registration or publication (Item 2, Table 2). The US Food and Drug Administration has introduced Diversity Action Plans for clinical trials (FDA‐2021‐D‐0789), while the European Medicines Agency has issued guidance emphasizing representativeness and subgroup analyses (EMA/CHMP/539146/2013). In the UK, NICE (ECD9) decision‐making depends upon evidence that is applicable to real‐world populations, which are likely to receive treatment within the NHS. Consequently, improving representation is not only an ethical imperative but is increasingly aligned with regulatory expectations. These developments provide a basis for operationalizing representation across the trial lifecycle, including prospective consideration of diversity in site selection and recruitment, prespecified subgroup analyses, regulatory submissions, and post‐marketing evaluation.

Finally, restrictive eligibility criteria continue to limit the inclusion of those with comorbidities. Exclusion criteria are often introduced to minimize safety risks, reduce confounding, improve the interpretability of efficacy signals, and satisfy regulatory requirements during early‐phase development. Nevertheless, excessive exclusion may compromise external validity. Future studies should seek an appropriate balance between internal validity and generalizability using pragmatic designs, stratification approaches, and post‐marketing studies where appropriate. In other words, whenever feasible from a safety and regulatory perspective, exclusion criteria that are not essential to a study's scientific objectives – particularly those related to common comorbidities – should be carefully re‐evaluated and minimized to broaden eligibility and enhance participant representation (Item 4, Table 2). This is important primarily in the context of underserved populations that experience a higher burden of chronic health conditions and comorbidities and may be disproportionately excluded by restrictive eligibility requirements.^54^ In parallel, standardized reimbursement for travel and caregiving costs, alongside decentralized or hybrid trial models, can help address financial and logistical barriers to participation. Reimbursement should be recognized as an equity measure rather than undue inducement and supported by clear regulatory guidance on fair compensation practices (Item 5, Table 2).

Despite these proposed strategies, structural barriers continue to contribute to the underrepresentation of diverse populations in AD clinical trials.^42^, ^55^ Strategies such as decentralized trial sites, participant engagement metrics, and community‐embedded recruitment show promise, but evidence showing their effectiveness across different settings and populations is limited. Important knowledge gaps therefore remain regarding how these approaches should be implemented and evaluated, how long‐term community partnerships can be sustained, and how accountability for diversity goals can be ensured across sponsors, research sites, and regulatory systems.

Of note, the recommendations presented here were derived from a single international roundtable and should not be interpreted as a formal consensus statement. Although informed by relevant literature and stakeholder perspectives, this manuscript does not represent a systematic review of the evidence. In addition, some perspectives may be underrepresented, including those from a broader range of LMICs, regulatory bodies, and community organizations. Future work should evaluate the feasibility and effectiveness of the proposed recommendations in diverse research settings.

Improving diversity in AD trials is both a scientific and moral urgency, where inclusion should be regarded as a measure of research quality, rather than an ancillary goal. By operationalizing inclusive engagement, transparent data sharing, fair reimbursement, and equitable eligibility criteria, stakeholders can collectively ensure that advances in AD clinical research will benefit all populations, not just those most easily reached.

## CONFLICT OF INTEREST STATEMENT

All disclosures are based on International Committee of Medical Journal Editors forms for the past 36 months. Igor C. Fontana, Tara Comber, Christine Chow, Tiffany W. Chow, Sebastian J. Crutch, Lynne Hughes, and Richard James Oakley have nothing to disclose. Sheena Aurora is a full‐time employee of the Alzheimer's Association, so all her travel for attending meetings and/or conferences is covered by her employer. Daniel J. Blackburn received i4i funding from the National Institute for Health and Care Research (NIHR) and consulting fees and honoraria for lectures from Eisai and Eli Lilly and participated in the ARUK Scientific Advisory Committee (unfunded). Rachel F. Buckley received funding from R01AG079142, DP2AG082342, and CureAlzheimer's Fund; honoraria for lectures from Karolinska Institute (invited lecture), UsAgainstAlzheimer's (CME Talk on Menopause and AD), UT Dallas (Invited lecture), The Transmitter Editorial; participated on the Women's Health Steering Committee, Sex and Gender In Neurodegeneration (S|GN) Consortium, Ann S. Bowers Women's Brain Health Institute; Chair of Sex & Gender ISTAART Professional Interest Area, co‐lead of S|GN consortium. Ruth Croney is an employee of and owns stock or stock options in F. Hoffmann‐La Roche Ltd. Karen Chilcott Holdridge is an employee of and owns stock or stock options in Eli Lilly and Company. Christopher Kipps received funding from the NIHR and UK Research and Innovation (UKRI); provided consultancy services to Eisai, Eli Lilly, Novartis, and Roche. Susan Kohlhaas is a member of the Alzheimer's Disease Data Initiative Advisory Board, of the UK Dementia Research Institute Board of Trustees, and of the Dementia Discovery Fund Scientific Advisory Board. Jorge Llibre‐Guerra received the following grants: K01AG073526, MJFF‐020770, AARFD‐21‐851415, 24HPE‐1287320, SG‐20‐690363, ALZSI‐25‐1464205, DAC‐GC‐02230426.01, and NIH‐1U01AG084514‐01A1. Paresh Malhotra received funding from the NIHR and ARUK in addition to UK Dementia Research Institute, Football Association (UK), FIFA, Medical Research Council, Alzheimer's Society, Lifearc; participated in data and safety monitoring boards, Johnson & Johnson boards and leadership on Alzheimer's Society Research Strategy Council and NIHR RDN National Specialty Lead for Dementia and Neurodegeneration and received a “drugs‐only” grant from Shire/Takeda Pharmaceuticals. Charles R. Marshall received funding from Innovate UK, Alzheimer's Research UK, Alzheimer's Society, NIHR, Tom and Sheila Springer Charity, Michael J. Fox Foundation; consulting fees from Eli Lilly, Eisai, and Roche; and leadership on NHS England. Stella McCaughey is a shareholder in Alector and Atsena Therapeutics. Rema Raman received funding from the NIA, Alzheimer's Association, American Heart Association, Eisai, and Eli Lilly (all grants to the institution) and participated on NIA and Trials Veterans Association Trial boards. Daniel Ridley holds stock in BMS and Biogen. Shardae Showell was supported by Melanie Shulman on the writing of this manuscript and is an employee of Biogen, in which she may hold stock options. Richard Tsai is an employee of Denali Therapeutics and may hold stock or stock options. Catherine J. Mummery received a grant from Biogen for an investigator‐led trial of ultrafast MRI in dementia; consulting fees from Biogen, Roche, Lilly, Eisai, Novartis, Neurimmune, MSD, and GSK; honoraria for lectures from Eisai and Lilly; and travel and accommodation support for attending meetings from Biogen, Eisai, Roche, and Alector. She served as a Lilly expert advisor in the development of a clinical program of siRNA J4T‐MCL‐0LAA, as a Lilly member of the advisory board on donanemab Trailblazer for which she received fees, as a member of the Novartis advisory board on an AD drug program steering committee for which she received fees, as a member of the Roche/Genentech advisory board for trontinemab for which she received fees, as a member of the Eisai advisory board on UK AUR for Leqembi for which she received fees, and as the chair of the data safety monitoring board for Immunobrain. She is Director of the NIHR UK Dementia Trials Network and co‐chair of NIHR dementia Translational Research Collaboration. Author disclosures are available in the Supporting Information.

## Supporting information




**Supporting Information**: alz71928‐sup‐0001‐TableS1.docx

**Supporting Information**: alz71928‐sup‐0002‐DisclosureForms.pdf
