# Supplementary material for: UK Dementia Trials Network: an expert‐informed framework for improving representation in dementia trials
Source: Alzheimers Dement. 2026 Oct 26;22(10):e71928. doi: 10.1002/alz.71928 (PMC13696990; doi:10.1002/alz.71928)
Supplement: Supplementary file 2 [file ALZ-22-e71928-s001.docx]

Supplementary Table 1

| **Name** | **Roundtable role** | **Affiliation** | **Expertise** |
| --- | --- | --- | --- |
| **Sheena Aurora** | Participant | Medical & Scientific Relations, Alzheimer's Association, Chicago, Illinois, USA | Alzheimer’s disease; clinical translation; healthcare implementation |
| **Daniel J. Blackburn** | Participant | Division of Neuroscience, School of Medicine and Population Health, Sheffield Institute for Translational Neuroscience (SITRaN), The University of Sheffield, Sheffield, UK | Dementia; digital cognitive assessment; diverse populations; speech/language biomarkers |
| **Rachel F. Buckley** | Panelist | Department of Neurology, Massachusetts General Hospital, Harvard Medical School, Boston, Massachusetts, USA; Melbourne School of Psychological Sciences, University of Melbourne, Melbourne, VIC, Australia | Alzheimer’s disease; sex & gender; biomarkers; dementia risk and progression |
| **Tara Comber** | Participant | Department of Neurodegenerative diseases, Institute of Neurology, University College London, Queen Square, London, UK | Clinical trial coordination; dementia research delivery; trial governance; research operations |
| **Christine Chow** | Participant | Department of Neurodegenerative diseases, Institute of Neurology, University College London, Queen Square, London, UK | Neurogenetic therapies; trial network operation; clinical research program management; translational research |
| **Tiffany W. Chow** | Moderator | Penn Neurodegeneration Genomics Center, Department of Pathology and Laboratory Medicine, University of Pennsylvania Perelman School of Medicine, Philadelphia, Pennsylvania, USA | Behavioral neurology; neuroimaging; dementia; neuropsychiatry assessment; clinical trial design; caregiver support |
| **Ruth Croney** | Panelist | Roche Products Ltd, Welwyn Garden City, UK | Dementia research; clinical research operations delivery; participant and patient engagement |
| **Sebastian J. Crutch** | Participant | Dementia Research Centre, Institute of Neurology, University College London, Queen Square, London, UK; Rare Dementia Support, Institute of Neurology, University College London, Queen Square, London, UK | Young-onset and rare dementias; neuropsychology; psychosocial support; community engagement |
| **Igor C. Fontana** | Participant | Department of Neurodegenerative diseases, Institute of Neurology, University College London, Queen Square, London, UK | Dementia clinical trials; research engagement and communications; international dementia research; low- and middle-income countries research |
| **Karen Chilcott Holdridge** | Participant | Eli Lilly and Company, Indianapolis, Indiana, USA | Dementia research; clinical trials; research delivery and participant engagement; regulatory affairs |
| **Lynne Hughes** | Participant | Global Alzheimer's Platform Foundation, Washington DC, USA | Dementia; clinical development; clinical trial design and strategy; trial operations; recruitment and participant engagement; neurocognitive assessment |
| **Christopher Kipps** | Participant | Wessex Neurological Centre, University Hospital Southampton NHS Foundation Trust, Southampton, UK | Dementia and cognitive disorders; clinical trials; research delivery and infrastructure; digital health and secure data environments |
| **Susan Kohlhaas** | Participant | Alzheimer's Research UK, Cambridge, UK | Dementia research strategy; research funding; policy; innovation and research translation |
| **Jorge Llibre-Guerra** | Panelist | Washington University in St. Louis, St. Louis, Missouri, USA | Dementia prevention; genetics; epidemiology; early biomarkers; clinical trials; low- and middle-income countries partnership; diverse populations |
| **Paresh Malhotra** | Participant | Department of Brain Sciences, Imperial College London, London, UK | Cognitive neurology; dementia; clinical trials; research delivery and implementation; biomarkers; novel trial design; NIHR research networks |
| **Charles R. Marshall** | Panelist | Centre for Preventive Neurology, Wolfson Institute of Population Health, Queen Mary University of London, London, UK; Department of Neurology, Barts Health NHS Trust, London, UK | Cognitive neurology; dementia; epidemiology; health inequalities and diverse populations |
| **Stella McCaughey** | Participant | Atsena Therapeutics, Durham, North Carolina, USA | Dementia research; clinical trials; participant engagement and research delivery |
| **Catherine J. Mummery** | Participant | Dementia Research Centre, Institute of Neurology, University College London, Queen Square, London, UK | Dementia clinical trials; clinical development; translational research; NIHR research networks; trial delivery and research infrastructure |
| **Richard James Oakley** | Participant | Alzheimer's Society, London, UK | Dementia research strategy; research funding; clinical research; policy and research translation |
| **Rema Raman** | Participant | Alzheimer's Therapeutic Research Institute, University of Southern California, San Diego, CA, USA | Biostatistics; clinical trial design and analysis; recruitment, engagement and retention; Alzheimer’s clinical trials; inclusivity |
| **Daniel Ridley** | Participant | Bristol Myers Squibb, New York, New York, USA | Dementia clinical research; trial delivery; participant engagement |
| **Shardae Showell** | Panelist | Biogen Inc, Cambridge, Massachusetts, USA | Community engagement; diversity and inclusion; dementia research participation |
| **Richard Tsai** | Participant | Denali Therapeutics, South San Francisco, California, USA | Dementia clinical research; clinical trials; research delivery |
